# Skeletal adaptations in young male mice after 4 weeks aboard the International Space Station

**DOI:** 10.1038/s41526-019-0081-4

**Published:** 2019-09-24

**Authors:** Kevin A. Maupin, Paul Childress, Alexander Brinker, Faisal Khan, Irushi Abeysekera, Izath Nizeet Aguilar, David J. Olivos, Gremah Adam, Michael K. Savaglio, Venkateswaran Ganesh, Riley Gorden, Rachel Mannfeld, Elliott Beckner, Daniel J. Horan, Alexander G. Robling, Nabarun Chakraborty, Aarti Gautam, Rasha Hammamieh, Melissa A. Kacena

**Affiliations:** 1 Department of Orthopaedic Surgery, Indiana University School of Medicine, Indianapolis, IN USA; 2 Richard L. Roudebush VA Medical Center, Indianapolis, IN USA; 3 Biochemistry and Molecular Biology, Indiana University School of Medicine, Indianapolis, IN USA; 4 Microbiology and Immunology, Indiana University School of Medicine, Indianapolis, IN USA; 5 Anatomy and Cell Biology, Indiana University School of Medicine, Indianapolis, IN USA; 6 U.S. Army Center for Environmental Health Research, Fort Detrick, MD USA; 7 Geneva Foundation, Fort Detrick, MD USA

**Keywords:** Translational research, Anatomy

## Abstract

Gravity has an important role in both the development and maintenance of bone mass. This is most evident in the rapid and intense bone loss observed in both humans and animals exposed to extended periods of microgravity in spaceflight. Here, cohabitating 9-week-old male C57BL/6 mice resided in spaceflight for ~4 weeks. A skeletal survey of these mice was compared to both habitat matched ground controls to determine the effects of microgravity and baseline samples in order to determine the effects of skeletal maturation on the resulting phenotype. We hypothesized that weight-bearing bones would experience an accelerated loss of bone mass compared to non-weight-bearing bones, and that spaceflight would also inhibit skeletal maturation in male mice. As expected, spaceflight had major negative effects on trabecular bone mass of the following weight-bearing bones: femur, tibia, and vertebrae. Interestingly, as opposed to the bone loss traditionally characterized for most weight-bearing skeletal compartments, the effects of spaceflight on the ribs and sternum resembled a failure to accumulate bone mass. Our study further adds to the insight that gravity has site-specific influences on the skeleton.

## Introduction

The Earth’s resources are finite and will continue to diminish as the human population continues to grow. Therefore, there is a great need to explore beyond our planet for colonization and resource acquisition. Currently, one of our biggest hurdles to accomplishing this, are the responses of our own physiology to extended exposure to outer space (e.g., the loss of bone and muscle mass due to the absence of loading from Earth’s gravity).^1–8^ Therefore, continued efforts need to be made to understand all of the physiological effects of spaceflight on living beings.

Although there is a growing body of literature regarding the negative impacts of spaceflight on bone health,^6,8–12^ the number of investigations remains small, and the variables assessed are limited (e.g., age, sex, species, strain, duration of spaceflight exposure, skeletal sites examined, types of analyses completed on skeletal tissues, etc.). Mice are currently the animal of choice for spaceflight investigations primarily due to their smaller size compared to rats, allowing for more animals/group within the same caging constraints and similarities to the human skeleton. Although spaceflight studies are generally not ideal in terms of experimental design, they remain critical to study bone physiology, which is necessary for the continued desire for space exploration by humans.

As part of a multi-institutional study, we had the opportunity to examine several bones from the skull (calvaria, mandible, and incisor), torso (L4 vertebrae, tenth rib, and the third body of the sternum), and limbs (humerus, tibia, and femur) from C57BL/6, male mice which were 9-weeks-old at the time of launch and remained in spaceflight for ~4 weeks. There were two control groups, mice housed in spaceflight hardware on Earth and mice, which were euthanized immediately after launch (baseline controls). With access to these specimens, we were able to test the hypothesis that weight-bearing bones would experience an accelerated loss of bone mass compared to non-weight-bearing bones, and that spaceflight would also inhibit skeletal maturation in male mice.

Importantly, while previous NASA studies were conducted with less aggressive female mice, here male mice were cohabitated in spaceflight, and thus one of the few times that the impact of spaceflight on the male mouse skeleton has been studied. Examination of male mice is particularly important as more than 89% of space explorers have been male (https://bigthink.com/think-tank/women-in-space-by-country). However, due to their aggressive behavior, few spaceflight missions have successfully flown male mice. Indeed, before our mission, male mice were flown on a Russian Bion-M1 biosatellite in groups of 3 and only 16 of 45 survived the spaceflight. Of the surviving 16 mice, 38% had tail injuries and 25% had limb injuries. Although the causes of these injuries were not clear, these results increased NASA’s concern for cohousing male mice in spaceflight until we completed our previously published preflight acclimation/behavior studies^13–15^ and until this successful mission. Recently, using JAXA spaceflight hardware, male mice were individually housed in spaceflight.^12^ Because aggression and singly housing mice both cause anxiety and stress which can affect the skeleton, previous observations in male mice must be viewed with this caveat in mind.

In addition, in the studies reported here, mice were 9-weeks-old at launch and stayed aboard the ISS for ~4 weeks. As skeletal maturity in mice occurs around 16–18 weeks, this study design allowed us to examine the effects of microgravity on skeletal maturation. In humans, for comparison, skeletal maturity is reached at ~30 years-of-age. According to NASA’s website (astronauts.nasa.gov/content/faq.html), the average age of current astronaut applicants is 34 years old, which puts them past skeletal maturity before training. Plans for long-term colonization of the Moon or Mars will likely involve younger astronauts and animals of social and ecological importance. Therefore, this skeletal survey also highlights some of the negative consequences that space will have on achieving peak bone mass in skeletally immature males.

## Results

The results from these studies are divided into two main groups: weight-bearing (limb bones: humerus, femur, and tibia as well as the L4 vertebrae) and non-weight-bearing bones (all skull and torso bones with the exception of the L4 vertebrae: calvaria, mandible, incisior, sternum, and tenth rib). For each skeletal site examined, we report significant and trending data for comparisons between spaceflight and ground samples as well as comparisons between baseline samples and ground or flight samples. The latter would reflect changes with maturation.

### Non-weight-bearing skeletal sites

**Fig. 1:**
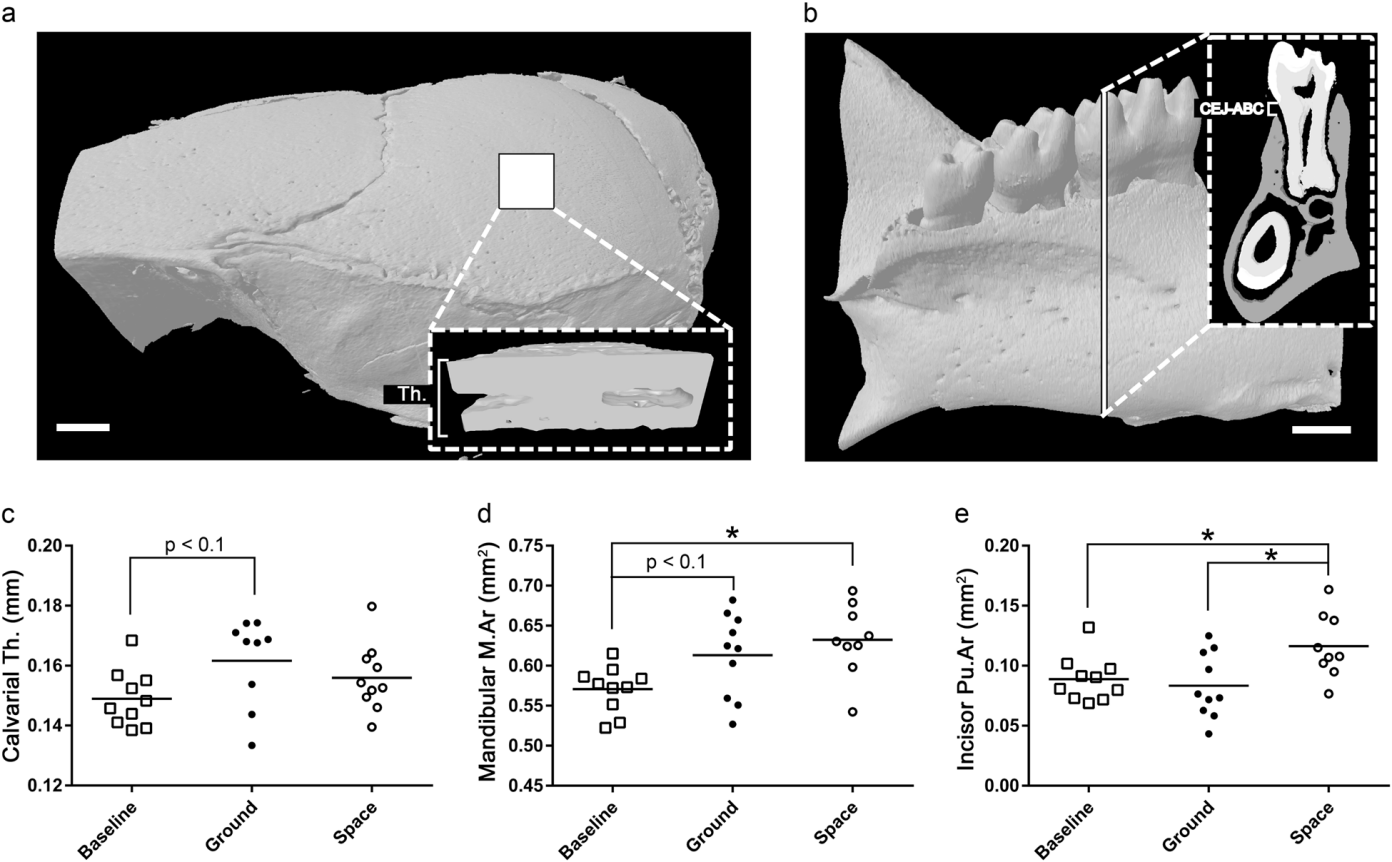
The effects of microgravity on the calvarium and mandible. Representative sectioning of the calvarium (*n* = 10, scale bar = 10 pixels) (**a**) and mandible (*n* = 10, scale bar = 1 mm) (**b**) for measurements of bone parameters by µCT. **c**–**e** Selected parameters of interest for calvarium (**c**), mandible (**d**), and incisor (**e**). Dots indicate measured value for a single animal. Black bars represent means. Group means compared by one-way ANOVAs with Holm–Sidak post-hoc analyses. *p < 0.05

There were no significant changes measured for the parietal bone of the mouse calvarium between ground and spaceflight samples (Table 1). There were, however, trends toward increased calvarial thickness (Fig. 1c) and bone volume (Table 1) in the ground control samples compared to baseline samples (11%; *p* < 0.1 and 7.8%; *p* < 0.1, respectively). These results likely represent maturation between euthanization at 9 weeks (baseline animals) and euthanization at 13 weeks (ground control animals).

**Table 1:** Bone mass parameters for the mouse skull as measured by µCT following 1 month on ground or in space

Variables compared	Baseline	Ground	Space	*p*-value (ground vs. space)
Calvarium (parietal)				
BV/TV (%)	96.23 ± 0.55	95.88 ± 0.96	97.71 ± 0.50	0.357
TV (mm^3^)	0.053 ± 0.001	0.058 ± 0.002	0.054 ± 0.001	0.395
BV (mm^3^)	0.051 ± 0.001	0.055 ± 0.002	0.053 ± 0.001	0.408
MV (mm^3^)	0.002 ± 0.001	0.003 ± 0.001	0.001 ± 0.001	0.313
Thickness (mm)	0.149 ± 0.003	0.165 ± 0.005	0.156 ± 0.004	0.352
Mandible				
B.Ar/T.Ar (%)	69.21 ± 0.44	68.71 ± 0.53	67.77 ± 0.66	0.415
T.Ar (mm^2^)	1.853 ± 0.021	**1.957** **±** 0.024**	**1.962** **±** 0.019**	0.880
B.Ar (mm^2^)	1.283 ± 0.018	**1.344** **±** 0.011*	1.330 ± 0.017	0.515
M.Ar (mm^2^)	0.571 ± 0.009	0.613 ± 0.017	**0.633** **±** 0.015*	0.342
CEJ–ABC (mm)	0.206 ± 0.006	0.204 ± 0.012	0.193 ± 0.008	0.673
Incisor				
[E + D]Ar/T.Ar (%)	82.06 ± 1.21	82.11 ± 1.87	**75.48** **±** 1.87*	**0.029**
T.Ar (mm^2^)	0.494 ± 0.003	**0.468** **±** 0.005***	**0.474** **±** 0.004**	0.259
[E + D]Ar (mm^2^)	0.406 ± 0.007	0.384 ± 0.011	**0.358** **±** 0.010**	0.120
Pu.Ar (mm^2^)	0.089 ± 0.006	0.083 ± 0.009	**0.116** **±** 0.009*	**0.021**

Values are expressed as mean ± S.E.M. (*n* = 10)

*p*-values calculated using one-way ANOVAs followed by Holm–Sidak post-hoc analyses. Bolded values to highlight significant *p*-values

*BV* bone volume, *TV* tissue volume, *MV* marrow volume, *B.Ar* bone area, *T.Ar* tissue area, *M.Ar* marrow area, *CEJ–ABC* cementoenamel junction to alveolar bone crest, *[E* *+* *D]Ar* [enamel + dentin] area, *Pu.Ar* dental pulp area

For comparisons to baseline: *p < 0.05, **p < 0.01, ***p < 0.001

For the mandible, following the subtraction of the molar and incisor, there were no significant differences observed between ground and spaceflight samples. However, there were significant increases in mandibular tissue area (T.Ar) in the ground control group compared to the baseline group (5.6%; *p* < 0.01), and in the spaceflight group compared to the baseline group (5.9%; p < 0.01). There was also a significant increase in the mandibular bone area (B.Ar) between the ground control group and the baseline group (4.8%; *p* < 0.05), and in spaceflight specimens compared to baseline controls (3.7%; *p* < 0.1). On the other hand, there was a significant increase in marrow area (M.Ar; Fig. 1d) for spaceflight samples compared to baseline controls (11%; *p* < 0.05), whereas there was a trending increase in ground samples compared to baseline controls (7.4%; *p* < 0.1).

Although spaceflight did not affect the calvarium or the mandible, spaceflight did affect the mouse incisor (spaceflight vs. ground specimens). Indeed, there was a significant decrease in the percentage of tissue area that was occupied by enamel and dentin ([E + D]Ar/T.Ar; Table 1; 8.0%; *p* < 0.05) associated with microgravity, which was primarily due to increased expansion of the dental pulp cavity (Pu.Ar; Fig. 1e; 30%; *p* < 0.05), which did not occur in the ground samples.

**Fig. 2:**
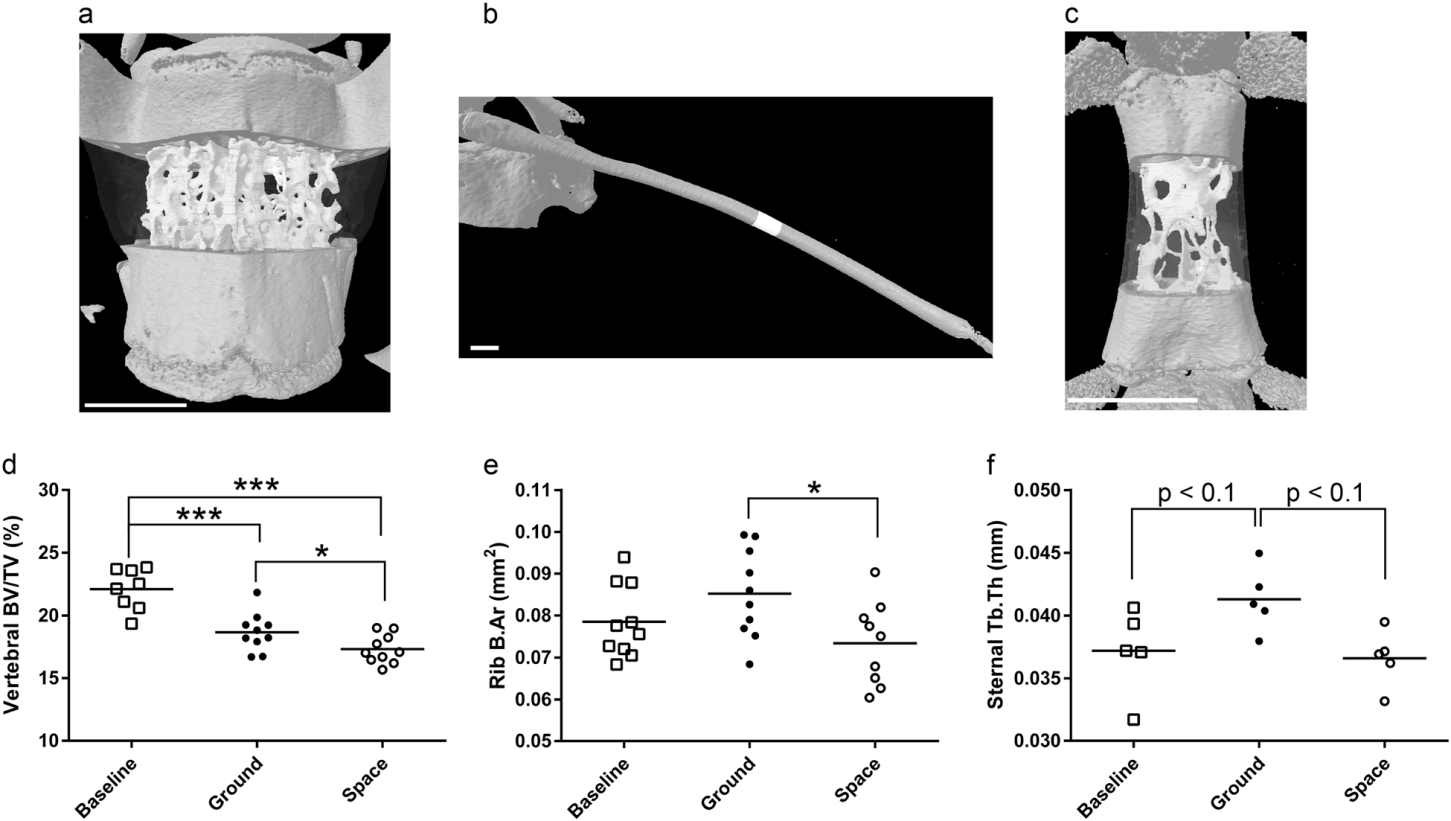
The effects of microgravity on the vertebrae, ribs, and sternebrae. Representative sectioning of the L4 vertebra (*n* = 10, scale bar = 1 mm) (**a**), tenth rib (*n* = 10, scale bar = 0.5 mm) (**b**), and third sternebra (*n* = 5, scale bar = 1 mm) (**c**) for measurements of bone parameters by µCT. **d**–**f** Selected parameters of interest for vertebrae (**d**), ribs (**e**), and sternebrae (**f**). Dots indicate measured value for a single animal. Black bars represent means. Group means compared by one-way ANOVAs with Holm–Sidak post-hoc analyses. *p < 0.05, ***p < 0.001

For the tenth rib, when comparing ground samples to baselines, there were trends (*p* < 0.2; Table 2) toward increased rib axial expansion: T.Ar (12%, *p* = 0.154) and M.Ar (25%, *p* = 0.162). Spaceflight rib samples showed no evidence of axial expansion, resulting in significantly lower B.Ar (14%; *p* < 0.05; Fig. 2e) compared to ground controls. There was also a significant reduction in cortical thickness in the spaceflight rib samples compared to baselines (7.1%; *p* < 0.05). These data suggest a delayed maturation in axial expansion and loss of the cortical shell associated with spaceflight.

**Table 2:** Bone mass parameters for the mouse torso as measured by µCT following 1 month on ground or in space

Variables compared	Baseline	Ground	Space	*p*-value (ground vs. space)
Vertebral body (L4)				
BV/TV (%)	22.11 ± 0.58	**18.68** **±** 0.48***	**17.31** **±** 0.36***	**0.043**
Tb.Th (mm)	0.054 ± 0.001	**0.049** **±** 0.002*	**0.047** **±** 0.001*	0.175
Tb.Sp (mm)	0.188 ± 0.003	**0.202** **±** 0.004*	**0.202** **±** 0.004*	0.954
Tb.N (mm^−1^)	4.062 ± 0.053	**3.811** **±** 0.056*	**3.714** **±** 0.087**	0.313
Rib (10th)				
B.Ar/T.Ar (%)	76.75 ± 1.40	74.19 ± 0.81	74.46 ± 0.87	0.862
T.Ar (mm^2^)	0.103 ± 0.004	0.115 ± 0.006	0.099 ± 0.005	0.087
B.Ar (mm^2^)	0.079 ± 0.003	0.085 ± 0.003	0.073 ± 0.003	**0.042**
M.Ar (mm^2^)	0.024 ± 0.002	0.030 ± 0.002	0.026 ± 0.002	0.277
Ct.Th (mm)	0.085 ± 0.002	0.083 ± 0.001	**0.079** **±** 0.001*	0.139
Sternebral body (3rd)				
BV/TV (%)	9.54 ± 0.35	9.68 ± 0.64	9.55 ± 0.71	0.998
Tb.Th (mm)	0.037 ± 0.002	0.041 ± 0.001	0.037 ± 0.001	0.061
Tb.Sp (mm)	0.214 ± 0.022	0.231 ± 0.009	0.189 ± 0.014	0.154
Tb.N (mm^−^^1^)	2.590 ± 0.171	2.354 ± 0.186	2.620 ± 0.222	0.694

Values are expressed as mean ± S.E.M.

*p*-values calculated using one-way ANOVAs followed by Holm–Sidak post-hoc analyses. Bolded values to highlight significant *p*-values

Vertebrae and ribs: *n* = 10. Sternebrae: *n* = 5. *BV* bone volume, *TV* tissue volume, *Tb.Th* trabecular thickness, *Tb.Sp* trabecular spacing, *Tb.N* trabecular number, *B.Ar* bone area, *T.Ar* tissue area, *M.Ar* marrow area, *Ct.Th* cortical thickness

For comparisons to baseline: *p < 0.05, **p < 0.01, ***p < 0.001

For the sternum, half of the baseline, ground, and spaceflight samples were saved to be processed for gene expression studies, leaving five samples available for micro-computed tomography (µCT) analyses, which decreased our power to detect statistically significant differences. We detected a trend toward increased trabecular thickness (Tb.Th) in the ground control animals compared to baselines (11%; *p* < 0.1; Fig. 2f); likely a result of skeletal maturation during the experiment. The increase in sternebrae Tb.Th did not occur in spaceflight samples, resulting in a trend toward a decrease in Tb.Th between spaceflight vs. ground samples (9.8%; *p* < 0.1; Fig. 2f).

### Weight-bearing skeletal sites

In the L4 vertebrae, skeletal maturation likely caused a significant reduction in bone volume/tissue volume (BV/TV) in both ground and space samples (Fig. 2d; 16% and 22%, respectively; *p* < 0.001) vs. baseline. Of note, the reduction in BV/TV was greater in the spaceflight vertebrae samples compared to ground samples (7.3%; *p* < 0.05). Tb.Th, trabecular number (Tb.N), and trabecular spacing (Tb.Sp) were all significantly lower in both spaceflight and ground samples compared to baseline vertebrae (Table 2). However, no significant differences were detected in Tb.Th, Tb.N, nor Tb.Sp between spaceflight and ground control vertebrae.

For the limbs, five femora and tibiae were available for µCT analyses. Again, half of the samples were sequestered for gene expression analyses. However, we did have the full ten samples available for analyzing the humeri.

**Fig. 3:**
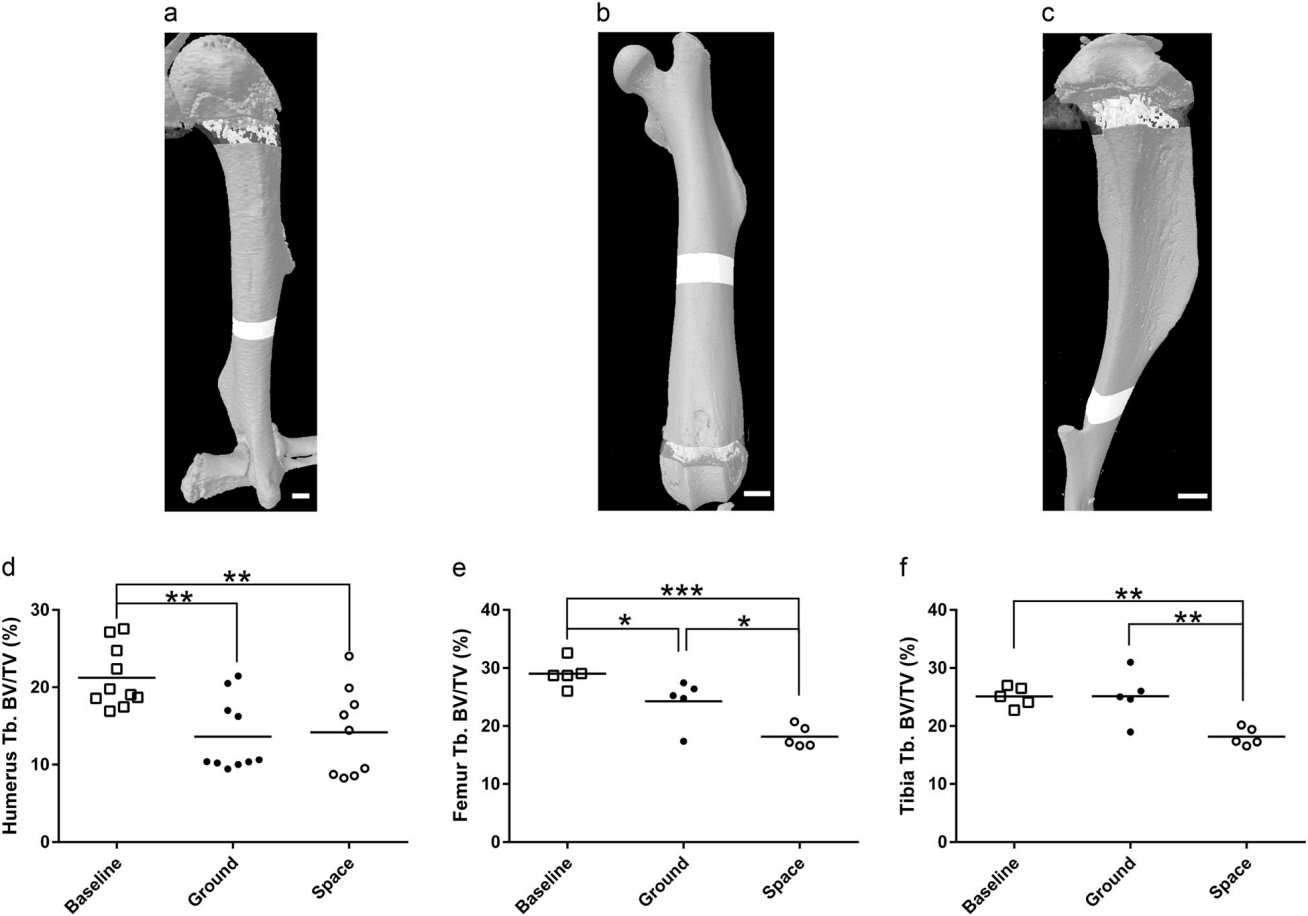
The effects of microgravity on the humerus, femur, and tibia. Representative sectioning of the humerus (*n* = 10, scale bar = 0.5 mm) (**a**), femur (*n* = 5, scale bar = 1 mm) (**b**), and tibia (*n* = 5, scale bar = 1 mm) (**c**) for measurements of bone parameters by µCT. **d**–**f** BV/TV measurements for proximal humerus (**d**), distal femur (**e**), and proximal tibia (**f**). Dots indicate measured value for a single animal. Black bars represent means. Group means compared by one-way ANOVAs with Holm–Sidak post-hoc analyses. *p < 0.05, **p < 0.01, ***p < 0.001

The only significant changes in bone parameters that we observed in the humerus were in trabecular bone and in response to maturation. When comparing ground samples to baseline, there was a sharp decline in BV/TV (36%; *p* < 0.01; Fig. 3d), which was due to losses in both Tb.Th (9.9%; *p* < 0.05) and Tb.N (29%; *p* < 0.01), as well as an increase in Tb.Sp (29%; *p* < 0.05). These results can be found in Table 3. Unlike the loss of trabecular architecture in the L4 verterbae, the degree of changes in the humeri spaceflight samples were approximately equal to those observed in ground samples, suggesting no additional influence of microgravity on bone loss.

**Table 3:** Bone mass parameters for the mouse limbs as measured by µCT following 1 month on ground or in space

Variables compared	Baseline	Ground	Space	*p*-value (ground vs. space)
Humerus (Tb.Proximal)				
BV/TV (%)	21.23 ± 1.26	**13.63** **±** 1.49**	**14.18** **±** 1.92**	0.804
Tb.Th (mm)	0.071 ± 0.002	**0.064** **±** 0.002*	**0.064** **±** 0.002*	0.890
Tb.Sp (mm)	0.189 ± 0.006	**0.244** **±** 0.013*	**0.232** **±** 0.016*	0.524
Tb.N (mm^−1^)	2.967 ± 0.123	**2.105** **±** 0.179**	**2.161** **±** 0.234**	0.831
Humerus (Ct.Midshaft)				
B.Ar/T.Ar (%)	60.31 ± 0.63	61.44 ± 0.64	60.47 ± 0.73	0.543
T.Ar (mm^2^)	0.957 ± 0.011	0.941 ± 0.022	0.917 ± 0.016	0.545
B.Ar (mm^2^)	0.577 ± 0.008	0.578 ± 0.016	0.554 ± 0.011	0.431
M.Ar (mm^2^)	0.380 ± 0.008	0.363 ± 0.010	0.362 ± 0.010	0.989
Ct.Th (mm)	0.187 ± 0.003	0.191 ± 0.004	0.185 ± 0.003	0.444
Femur (Tb.Distal)				
BV/TV (%)	29.02 ± 1.05	**24.24** **±** 1.77*	**18.17** **±** 0.84***	**0.012**
Tb.Th (mm)	0.057 ± 0.002	0.054 ± 0.002	**0.048** **±** 0.002*	0.113
Tb.Sp (mm)	0.125 ± 0.001	**0.144** **±** 0.004**	**0.154** **±** 0.003***	**0.030**
Tb.N (mm^−1^)	7.069 ± 0.041	**6.352** **±** 0.084***	**6.039** **±** 0.099***	**0.016**
Femur (Ct.Midshaft)				
B.Ar/T.Ar (%)	46.52 ± 1.67	45.73 ± 0.859	44.53 ± 0.786	0.734
T.Ar (mm^2^)	2.074 ± 0.078	2.138 ± 0.135	2.077 ± 0.062	0.957
B.Ar (mm^2^)	0.962 ± 0.034	0.973 ± 0.046	0.924 ± 0.018	0.700
M.Ar (mm^2^)	1.112 ± 0.068	1.164 ± 0.090	1.154 ± 0.048	0.940
Ct.Th (mm)	0.218 ± 0.008	0.216 ± 0.003	0.207 ± 0.003	0.442
Tibia (Tb.Proximal)				
BV/TV (%)	25.07 ± 0.78	25.11 ± 1.91	**18.18** **±** 0.69**	**0.006**
Tb.Th (mm)	0.052 ± 0.001	0.052 ± 0.001	**0.045** **±** 0.003*	0.056
Tb.Sp (mm)	0.126 ± 0.002	0.130 ± 0.005	**0.154** **±** 0.007**	**0.015**
Tb.N (mm^−1^)	7.070 ± 0.113	6.897 ± 0.200	**5.965** **±** 0.353*	**0.038**
Tibia (Ct.Midshaft)				
B.Ar/T.Ar (%)	69.30 ± 0.81	70.67 ± 1.11	69.59 ± 1.67	0.835
T.Ar (mm^2^)	1.056 ± 0.022	1.034 ± 0.046	1.025 ± 0.034	0.979
B.Ar (mm^2^)	0.732 ± 0.018	0.731 ± 0.036	0.715 ± 0.039	0.912
M.Ar (mm^2^)	0.324 ± 0.010	0.303 ± 0.017	0.310 ± 0.011	0.715
Ct.Th (mm)	0.259 ± 0.005	0.263 ± 0.009	0.257 ± 0.013	0.961

Values are expressed as mean ± S.E.M.

*p*-values calculated using one-way ANOVAs followed by Holm–Sidak post-hoc analyses. Bolded values to highlight significant *p*-values

Humeri: *n* = 10. Femora and tibiae: *n* = 5. *BV* bone volume, *TV* tissue volume, *Tb.Th* trabecular thickness, *Tb.Sp* trabecular spacing, *Tb.N* trabecular number, *B.Ar* bone area, *T.Ar* tissue area, *M.Ar* marrow area, *Ct.Th* cortical thickness

For comparisons to baseline: *p < 0.05, **p < 0.01, ***p < 0.001

In the femur, we also failed to detect any significant changes in cortical bone (Table 3); however, there were several notable changes in trabecular bone mass. As shown in Fig. 3e, maturation significantly decreased trabecular BV/TV in the femora of the ground mice compared to baseline mice (17%; *p* < 0.05), this was exacerbated by spaceflight (37%; *p* < 0.001). When compared to ground samples, spaceflight femora showed significant reductions in BV/TV (25%; *p* < 0.05) and Tb.N (4.9%; *p* < 0.05), as well as a significant increase in Tb.Sp (6.9%; *p* < 0.05). There was also a trend toward reduced Tb.Th in spaceflight femora compared to ground control femora (11%; *p* = 0.113). These results can be found in Table 3.

Consistent with results in the humeri and femora, we observed no significant effect of maturation or spaceflight on the cortical parameters of the tibia. A unique finding in the trabecular bone in the tibia was that we observed no significant differences between ground and baseline samples, but we did observe significant reductions in trabecular bone in the spaceflight samples (Table 3) when compared to both baseline and ground samples. When compared to ground tibiae, spaceflight resulted in a 28% decrease in BV/TV (*p* < 0.01; Fig. 3f) and a 14% decrease in Tb.N (*p* < 0.05), as well as an 19% increase in Tb.Sp (*p* < 0.05). There was also a decrease in spaceflight tibia Tb.Th compared to that observed in ground tibia (14%), which just missed the cut-off for statistical significance (*p* = 0.056).

## Discussion

Although it has been more than 50 years since Soviet cosmonaut Yuri Gagarin became the first human to travel into space, the impact of spaceflight on human physiology remains an understudied area of investigation. That said, it is well known that spaceflight results in a loss of bone mass of weight-bearing bones such as the femur, tibia, and vertebrae. However, few non-weight-bearing bones have been analyzed to determine whether spaceflight differentially impacts skeletal sites. While published studies have examined the impact of spaceflight on a few sites of the mouse skeleton, the data is limited, and making comparisons between studies is challenging due to differences in the selection of mouse strain, age, sex, and experimental design (i.e., the use of different hardware, periods of microgravity exposure, or even whether the mice were euthanized in spaceflight or on the Earth after spaceflight where bone loading was re-established). In addition, the majority of human spaceflight data comes from males (89% of astronauts are men) whereas the majority of mouse spaceflight data comes from female mice, especially from NASA-supported missions (primarily due to aggressive behavior typically observed with male mice).^13,16^ On the basis of these observations, here we completed a skeletal survey of weight-bearing and non-weight-bearing bones in male C57BL/6 mice which were 9 weeks of age at launch and spent ~1 month in spaceflight.

With regard to non-weight-bearing sites, we examined the calvaria, mandible, incisor, ribs, and sternum. No changes were observed between spaceflight and ground control specimens for the calvaria, mandible or sternum. Our calvaria data (Table 1 and Fig. 1c) are consistent with that of Macaulay et al.^17^, showing there were no significant effects of 30 days of spaceflight (Russia’s Bion-M1 mission) on calvarial bones obtained from ~20-week-old male mice. This latter study is similar to our study in regard to sex and duration of flight; however, it was performed with slightly older, and therefore skeletally mature, mice. On the other hand, Zang et al.^18^ reported that on NASA’s STS-131 mission, 23-week-old female mice had increased calvarial bone volume, with a trend toward increased skull thickness following 15 days in microgravity. While further studies will be needed to confirm these findings, these limited data sets, suggest that sex-based differences may exist with respect to the impact of spaceflight on the calvarium.

With respect to the mandible, we did not observe differences in BV/TV or CEJ–ABC distance in spaceflight vs. ground control mice (Table 1 and Fig. 1d). This is consistent with a previous study of 9-week-old female mice, which were examined following 13 days, aboard NASA’s STS-135 space shuttle mission.^19^ However, 15 days of spaceflight resulted in a significant decrease in BV/TV in the mandible as well as a decrease in the CEJ–ABC distance in 23-week-old female mice flown on STS-131 mission, which is suggestive of altered tooth eruption.^19^ Unfortunately, with the limited spaceflight data available, it is unclear whether maturation alone or other factors are the cause of these apparently contradictory data sets.

The incisor was the only bone within the skull that we analyzed and found a significant difference between spaceflight and ground specimens. Specifically, we found a significant expansion of the dental pulp area (Table 1 and Fig. 1e); however, it is unclear whether this was due to changes in incisor eruption in spaceflight or altered tooth morphology. Of relevance, in the 9-week-old female mice exposed to spaceflight for 13 days on ST-135, it was noted that incisor length significantly increased compared to ground controls.^20^ With only two studies reporting incisor parameters, and with marked differences in experimental design (i.e., sex and age of mice as well as duration of spaceflight) it is difficult to determine why differences exist, but it does appear that spaceflight may impact incisors, at least in growing mice.

For both the ribs and sternebrae, we are unaware of other studies examining these bones, and therefore, we cannot make direct comparisons to other studies. However, these both represent non-weight-bearing bones that likely receive the majority of their mechanical loading from the action of breathing. For the ribs we observed a significant reduction in B.Ar (Fig. 2e) when comparing our spaceflight to ground samples and a non-significant trend toward reduced T.Ar and Ct.Th (Table 2). Because these values trended toward increasing in the ground, but not space samples, this is consistent with a reduced maturation-related cortical expansion. No significant differences were detected due to the effects of gravity on the sternum. However, we did observe a non-significant trend toward reduced Tb.Th in spaceflight (Table 2 and Fig. 2f), which may have reached statistical significance with more samples (*n* = 5). To verify whether there are true differences in either rib or sternum parameters will require further studies.

Although spaceflight resulted in limited or no changes in non-weight-bearing bones, marked differences were observed in the trabecular bone fraction in the L4 vertebrae, femur, and tibia (Tables 2 and 3 and Figs 2 and 3). On the other hand, no significant differences were observed in the trabecular bone within the humerus. Our trabecular bone data for lumbar vertebrae, femora, and tibiae are consistent with numerous spaceflight investigations using male and female mice of multiple ages over various mission durations. For example, in the L4 vertebrae, we observed similar changes (Fig. 2d and Table 2) to those reported for the male mice from the Bion-M1 mission where 30 days of spaceflight resulted in reduced bone mass in the vertebrae.^21,22^

With regard to the trabecular bone parameters of the tibia, we observed a significant decrease in trabecular BV/TV with no changes in cortical bone parameters (Fig. 3f and Table 3). Likewise, a study published very recently by Tominari et al.^12^ found a significant reduction in trabecular BV/TV with no differences in cortical bone parameters. Of note, the Tominari study design was very similar to ours. They examined the tibia of 9-week-old, male C57BL/6 mice exposed to spaceflight for 34 days. The main difference was the type of spaceflight hardware utilized. In addition, the tibia results from both studies are also consistent with what has been reported in 9-week-old females in the STS-108 mission.^23^

Similar to our studies (Fig. 3e and Table 3), reductions due to spaceflight in femoral trabecular bone fraction have been found by others. Specifically, another JAXA mission (MARS mission) in which 8-week-old male mice were subjected to spaceflight for 36 days showed a significant decrease in femoral trabecular bone mass.^24^ This was again observed in ~20-week-old male mice aboard the Bion-M1. In this study by Gerbaix et al., histological analysis demonstrates that osteoblast surface/bone surface is reduced while osteoclast surface/bone surface is elevated in spaceflight mice, explaining the reduction in bone mass. However, unlike in our study, the Bion-M1 study also showed that cortical thickness was decreased.^22^ Similarly, in 9-week-old female mice (STS-108), dynamic histomorphometry showed that cortical bone mass was reduced due to significant reductions in bone formation rates, indicative of reduced osteoblast activity.^23^

With regard to the humerus, we found no differences in either cortical or trabecular bone properties (Table 3 and Fig. 3d). Studies on the effects of spaceflight on the humerus in mice are limited, but in 9-week-old female mice aboard NASA’s STS-108 mission, 12 days of spaceflight had no significant effect on humerus bone mass.^23^ To our knowledge, aside from our data reported here on humeri, the only study reporting the effects of spaceflight on male mice humeri is the similarly designed work by Tominari et al.^12^ They found that, similar to the tibia, the spaceflight humeri exhibited a robust decline in trabecular bone mass. This is in stark contrast to our findings of no detectable changes in the trabecular bone of the humerus. As mentioned above, the main difference between these studies is the spaceflight hardware. In the Tominari study^12^ mice were individually housed in a much smaller space. Although cage dimensions are not provided, review of their provided videos show limited space for the mice to move. By contrast, the hardware used in our mission allowed mice to move throughout a large cage volume in spaceflight. Although not released by NASA, videos observed by several team members (MAK, PC, NC, and RH), show spaceflight mice pushing off with their forelimbs of the cage to move from one spot to the other. Thus, we suspect the differences between studies with respect to humeri data may be explained by this “loading” of the humerus in NASA hardware in the absence of gravity. In addition, mice are social animals and singly housing them is considered a source of stress, which could contribute to bone loss in multiple compartments including the humerus.^25,26^ Notably, STS-108 used an earlier version of the NASA Habitat used in our study, which may explain why their humeri data is consistent with ours.

Because we included baseline controls, we had the opportunity to examine how maturation (from 9 weeks to ~13 weeks) differs on the ground and in spaceflight. Studying the effects of spaceflight on skeletally immature mice is important as most studies focus on the mature skeleton. Not surprisingly, spaceflight had negative effects on the immature male skeleton. Interestingly, the negative effects of spaceflight on each skeletal compartment were not always driven by an enhanced loss of bone (e.g., vertebrae, tibia, femur, and humerus), but sometimes an apparent failure to accumulate bone mass (e.g., sternum) and/or undergo age-related bone modeling (e.g., ribs). As seen in “Spaceflight” column of Tables 1–3, all of the bone parameters reported for the incisor, along with the trabecular bone parameters for all weight-bearing bones (L4 vertebrae, tibia, femur, and humerus) were significantly different in spaceflight bones compared to baseline bones. Of importance, when examining changes between ground and baseline samples, for the L4 vertebrae, femur, and humerus, changes were in the same direction as that seen between spaceflight and baseline samples. However, for the tibia and for all but one parameter of the incisior (i.e., T.Ar), no changes were detected between ground and baseline samples. Taken together, our results regarding the effects of spaceflight on the natural skeletal maturation process reveal envelope-specific delays and could have wide-ranging implications, including for fracture healing which can employ pathways reminiscent to development.

## Methods

### Animals

7-week-old male C57BL/6J mice, purchased from Jackson Laboratories, were “Allo-reared” (cagemated from weaning) and maintained in their initial cohorts of 15 mice in “large” mouse cages (Ancare N40, polycarbonate, 19″ × 10.5″ × 6.125″) until they were placed into their final hardware/group. A full detailed description of the experimental and housing conditions have been previously published as part of preliminary experiments.^13–15^ Briefly, upon arrival to Kennedy Space Center (KSC), mice were housed in cohorts of 15 mice in N40 cages which contained a raised wire floor (3 openings/inch). This flooring was used to acclimate the mice to the metal wire caging on all six sides of the spaceflight hardware (i.e., the “Transporter” which houses the mice while they are on the SpaceX Dragon, and the “Habitat” which houses the mice while they are on the International Space Station or ISS (https://www.nasa.gov/ames/research/space-biosciences/rodent-research-hardware)). Pictures and detailed information related to NASA’s spaceflight hardware is provided by NASA (https://www.nasa.gov/sites/default/files/atoms/files/rodenthabitatsfs-13mar18.pdf). The footprint of the Transporter and Habitat are both 59.7 in^2^. By contrast, the N40 cage footprint is 199.5 in^2^. However, as the NASA hardware contains the wire mesh on all surfaces, the mice can climb and access virtually the entire interior surface area, which NASA refers to as the habitable surface area. The habitable surface area of the Transporter and Habitat are 715 and 882 in^2^, respectively. Of note, each side of the Transporter can house 10 mice while each side of the Habitat can house 5 mice. Currently, the SpaceX Dragon can accommodate 2 Transporters, housing up to a total of 20 mice/Transporter or 40 mice total. The ISS can currently accommodate 4 Habitats housing up to a total of 10 mice/Habitat or 40 mice total.

Upon arrival at KSC, mice were provided with water bottles containing a modified lixit which works identically to the lixits used in the spaceflight hardware. These lixits require more force that typical lixits and therefore some mice which could not utilize this hardware were excluded. Additionally mice were also acclimated to the NASA Nutrient-upgraded Rodent Food Bar (NuRFB) used in spaceflight. To confirm mice adapted to the utilization of the specialized lixit and the NuRFB, they were weighed twice weekly. Mice with a >10% weight loss were excluded.

N40 cages containing 15 mice were randomly assigned into groups. For this study, the groups were: flight, ground, and baseline (*n* = 10 per group). Two days before launch, the 10 “healthiest” mice per group were transferred from the N40 cages into Transporters (Flight and Ground groups) or were placed into new N40 cages for the baseline control group. It should be noted that “healthiest” was based upon a visual inspection of all 15 mice within an individual cage and was determined by NASA veterinarians (in collaboration with MAK and PC). Due to limited spaceflight hardware (no additional Transporters were available to house baseline control mice), baseline control mice remained in N40 cages from the time that the 10 healthiest mice from each group were selected, until they were euthanized at the time of launch (~2 days of different caging).

**Fig. 4:**
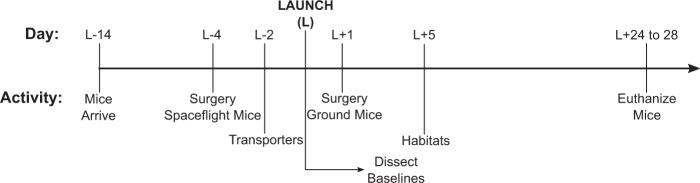
Experimental timeline. Launch timeline depicting the major events leading up to launch, launch, and concluding with mice euthanasia. Mice arrived to the Kennedy Space Center 2 weeks before launch to acclimate to spaceflight hardware. Four days before launch, surgeries or sham surgeries were performed. Two days before launch, the mice were loaded into spaceflight hardware (Transporters) and loaded onto the SpaceX Dragon Capsule. Launch occurred on February 19, 2017 at 14:39:00 UTC. Immediately after launch, baseline mice were euthanized. One day after launch asynchronous ground controls underwent surgery. Five days post launch, the mice were moved from the Transporter hardware into Habitats where they would remain for just under a month (ground control timeline duration was identical but was shifted to the right 5 days). Mice were euthanized between L + 24 and L + 28 days. Only the skeletal phenotypes from the sham surgery group are reported in this study

Figure 4 is a timeline related to the entire study. Notably, because this was a multi-institutional/agency study, we did not have access to all tissues for all types of analyses. Here we report the skeletal phenotype of wild-type C57BL/6 mice which served as untreated sham control animals from the larger parent experiment that addressed fracture healing in spaceflight. Mice were housed in spaceflight aboard the ISS as part of NASA’s Rodent Research 4 mission, which launched from KSC aboard SpaceX CRS-10 on February 19, 2017. It should be noted that sham here refers to mice which were anesthetized with ketamine/xylazine (90–125/10–20 mg/kg) and given buprenorphine (0.05 mg/kg, once at the time of surgery) just as the surgical mice, but no incision was made nor internal tissue manipulated, although wound clips were placed along the imaginary incision line located above the quadriceps. It should be noted that ground controls were asynchronous by 5 days to allow time for conditions aboard the ISS to be replicated on Earth for the ground controls. This includes environmental conditions such as cage temperature, timing of food and water changes, and time from euthanization to placement of dissected specimens into the cold stowage/freezers.

Mice were 9-weeks-old at launch and ~13-weeks-old at euthanization. Mice were euthanized by injection of ketamine/xylazine (150/45 mg/kg), followed by a closed chest cardiac puncture and cervical dislocation. For five mice in each group (including the sham mice evaluated here), the right hindlimb was removed at the hip, placed in 10% neutral buffered formalin (NBF), and transferred to 4 °C at the end of the day (approximately 4–6 hours later). These specimens remained at 4 °C in 10% NBF until they were returned to Indiana University School of Medicine ~2 weeks later. Then the samples were washed with ice cold phosphate buffered saline (PBS), transferred into ice cold 70% ethanol, and then stored at 4 °C until they were underwent µCT imaging as detailed below.

After removal of the right hindlimb, the remaining carcass was wrapped in aluminum foil and immediately transferred to the −95 °C cold stowage unit aboard the ISS (on Earth transferred to the −80 °C freezer). For the other 5 mice in each group, the carcass remained intact and the whole carcass was wrapped in aluminum foil and frozen as above. In preliminary studies conducted by NASA personnel on Earth, it took ~20 min for the middle of the carcass to reach −20 °C once placed in the −95 °C cold stowage unit or −80 °C freezer (MAK, personal communication). Carcasses remained at −80 °C or below from the time of insertion into cold stowage/freezer until they were shipped to Fort Detrick ~2 weeks later. The mice were then partially thawed (removed from freezer but on ice blankets) for ~15 minutes and tissue dissection was completed for the carcass (~45 min total out of −80 °C) and all bones examined here were immediately snap frozen with the exception of the humerus. The frozen bone specimens were then shipped to Indiana University School of Medicine and stored at −80 °C until they were removed for specific dissection as detailed below. For the humerus, after removal from the previously frozen carcass, the left whole foreleg was placed in 10% NBF for 72 h, then the samples were washed with ice cold PBS, transferred into ice cold 70% ethanol, shipped to Indiana University School of Medicine on water ice (~4 °C), and then stored at 4 °C until they were underwent µCT imaging. Of importance, all processing of frozen carcasses was done at the same time and identically for flight, ground, and baseline specimens.

Mice were euthanized between launch plus 24–28 (L + 24 and L + 28) days as astronauts could only euthanize and dissect 8 mice/day. All mice were maintained in accordance with the NIH Guide for the Care and Use of Laboratory Animals, whose experimental protocol was approved by the NASA Animal Care and Use Committee (protocol FLT-15-101/NAS-15-105).

### Micro-computed tomography

All of the skull and torso bones analyzed here, were obtained from previously frozen carcasses and were processed as follows. Samples were briefly thawed, defleshed and a standardized anatomical location for measurements (region of interest; ROI) was isolated if needed (e.g., ½ of the calvarium, ½ of the mandible, specific rib, specific vertebra, etc. as detailed below) and fixed in 10% neutral buffered formalin for 72 h, washed with ice-cold PBS, and then stored in ice-cold 70% ethanol at 4 °C. Of note, all processing of frozen tissue was done at the same time and identically for flight, ground, and baseline specimens. Calvariae, mandibles, ribs, vertebrae, sternums, and humeri were imaged using a desktop SkyScan 1172 µCT imaging system (SkyScan, Kontich, Germany) with all scans obtained at 60 kV using a 5.9 μm voxel size, other than the ribs, which were obtained at 9.8-μm voxel size. For all bones analyzed on the SkyScan, the higher threshold was 255. The following details the lower thresholds. For calvariae, ribs, and sternum the lower threshold was 90. For trabecular bone within the humerus, the lower threshold was 80. For the vertebrae and cortical bone of the humerus, the lower threshold was 110. For the mandible, the lower threshold was 120. Of note, the lower threshold was set to achieve a physiologically accurate representation of each bone. Images of each specimen were reconstructed with NRecon v.1.7.3. Bone structure parameters were visualized and determined using Skyscan software, Dataviewer, CTAn (Kontich, Belgium). Femora and tibiae were imaged using a desktop SCANCO µCT35 imaging system (SCANCO Medical, Brüttisellen, Switzerland) with all scans obtained at 55 kV using a 12 μm voxel size. For the trabecular bone analysis of the femora and tibiae, the lower threshold was 31 and the upper threshold was 500. For total bone and midshaft analyses, the lower threshold is 240 and the upper threshold is 700. Representations of selected areas used for analyses can be found in Figs 1–3. Although two different machines were utilized to complete µCT imaging and analyses, each skeletal site was only scanned on one machine, making comparisons possible without being concerned about variation between machines (e.g., the SCANCO system was used for all femora in all experimental groups).

### Individual ROI

The calvarial ROI was obtained by taking a 100 pixel^3^ volume that was centered on the parietal eminence. A 3D analysis was performed to obtain tissue volume (TV) and bone volume (BV). The fractional bone volume was calculated as BV/TV, and the marrow volume (MV) as TV-BV.[1] Calvarial thickness was determined by taking 3 thickness measurements from 3 random images within the ROI.

The mandibular ROI was defined as the cross-section of a single coronal slice through the middle of the posterior root of the first molar. After subtracting the molar from the region of interest, a 2D analysis was performed on the mandible with the incisor. A separate 2D analysis was also performed on the incisor alone. The reported mandible values (tissue area, T.Ar; bone area, B.Ar; marrow area, M.Ar = T.Ar – B.Ar [2]) were obtained by subtracting the equivalent incisor only values (T.Ar; enamel + dentin area, [E + D]Ar; and pulp area, Pu.Ar = T.Ar - [E + D]Ar [3]). The shrink-wrap function was used for both the mandible and incisor ROIs to insure accurate T.Ar measurements. The lingual cementum–enamel to alveolar bone crest distance (CEJ–ABC) was obtained by measuring the distance from the cementum edge on the lingual tooth surface to the alveolar bone apex.

For trabecular analyses of the vertebrae and sternum, ROIs which is also the TV, were obtained from 1 mm-tall-segments centered in the L4 vertebral and third sternebral bodies (term used to identify sternum when it is segmented into four sections), excluding the cortical bone. A 3D analysis was performed and reported variables include BV/TV where BV is calculated by the software during segmentation as the white area, trabecular thickness (Tb.Th), trabecular number (Tb.N), and trabecular spacing (Tb.Sp).

The reported rib cortical measurements include: T.Ar, B.Ar, B.Ar/T.Ar, M.Ar, and cortical thickness (Ct.Th). These values were obtained from a 2D analysis of a 0.5 mm ROI obtained from the midshaft of the tenth rib.

For trabecular analysis of the humeri, the ROI started at 0.5 mm distal from the proximal growth plate and extended an additional 0.5 mm distally. A 3D analysis was performed to obtain measurements for BV/TV, Tb.Th, Tb.N, and Tb.Sp. For the cortical analysis of the humeri, the ROI was set at 0.5 mm proximal from the midshaft and extended an additional 0.5 mm proximally, in order to avoid the deltoid tuberosity. A 2D analysis was performed to obtain T.Ar, B.Ar, B.Ar/T.Ar, M.Ar, and Ct.Th.

For trabecular analysis of the femur, the ROI started at 0.25 mm proximal of the distal growth plate and extended an additional 0.5 mm proximally. Reported variables include: BV/TV, Tb.Th, Tb.N, and Tb.Sp. For cortical analysis of the femur, a 1 mm ROI was centered on the midshaft of the femur. Initial variables obtained from the SCANCO software were BV and Ct.Th. BV was converted to B.Ar by dividing by the height of the analyzed bone segment (1 mm) and then T.Ar and M.Ar were calculated by the equation for the area of a cyclinder: B.Ar = π*(total radius^2^ − marrow radius^2^) [4]. By substituting total radius = Ct.Th + marrow radius and solving for marrow radius, we obtained the following equation: marrow radius = ((B.Ar/π)-Ct.Th^2^)/(2*Ct.Th) [5]. From there, M.Ar was calculated as π*marrow radius^2^, and T.Ar was calculated as M.Ar + B.Ar [6].

For trabecular analysis of the tibia, the ROI started at 0.25 mm distal of the proximal growth plate and extended an additional 0.5 mm proximally. Reported variables include: BV/TV, Tb.Th, Tb.N, and Tb.Sp. For cortical analysis of the tibia, a 1 mm ROI was obtained from a region that was 0.25 mm proximal from the tibiofibular junction. The cortical variables for tibiae were obtained similarly as those obtained from the femora.

### Statistics

One-Way ANOVAs followed by Holm–Sidak post-hoc analyses were used to compare bone parameters between baseline, space, and ground control samples. A significance threshold was set at *α* = 0.05. All statistical analyses and graphs were generated using Prism v6.07 (GraphPad, San Diego, CA) and figures were generated using InkScape (https://inkscape.org) or Adobe Photoshop (Adobe, San Jose, CA). Spaceflight treatment groups were limited to a maximum of 10 bones/site, except for the femur, tibia, and sternum, which were further limited to a maximum of 5 bones/site. We therefore discuss data as “trending” for changes associated with a *p*-value between 0.05 and 0.20. Presentation of all the data generated was deemed important for future inquiry.

### Reporting summary

Further information on research design is available in the Nature Research Reporting Summary linked to this article.

## Supplementary information

Reporting Summary

## Data Availability

The data that support the findings of this study are available from the corresponding author upon reasonable request.

## Supplementary information

**Supplementary information** accompanies the paper on the *npj Microgravity* website (10.1038/s41526-019-0081-4).
